# Bubble Trouble: Portal Venous Gas Embolism Following Hydrogen Peroxide Enema

**DOI:** 10.14309/crj.0000000000001882

**Published:** 2025-10-30

**Authors:** Prachi Mann, Tareq Alsaleh, Nouman Shafique, Mohamad Khaled Almujarkesh, Baha Aldeen Bani Fawwaz, Irteza Inayat

**Affiliations:** 1 Division of Internal Medicine, AdventHealth Orlando, Orlando, FL; 2 Division of Gastroenterology and Hepatology, AdventHealth Orlando, Orlando, FL

**Keywords:** hydrogen peroxide toxicity, enema adverse effects, chemical colitis, portal venous gas, hyperbaric oxygen therapy

## Abstract

Hydrogen peroxide (H_2_O_2_) is a caustic agent capable of causing serious gastrointestinal injury, yet its use as an enema is promoted in some alternative medicine communities for constipation. We report a case of a 56-year-old man who developed portal and mesenteric venous air embolism and chemical colitis following self-administration of an H_2_O_2_ enema. Imaging revealed extensive portal venous gas and colitis. Colonoscopy confirmed diffuse mucosal injury, and biopsies showed acute colitis with ulceration. The patient was treated with intravenous fluids, broad-spectrum antibiotics, and hyperbaric oxygen therapy, resulting in resolution of gas embolism. This case highlights the dangers of H_2_O_2_ enemas and the potential role of hyperbaric therapy in management.

## INTRODUCTION

Hydrogen peroxide (H_2_O_2_) is a potent oxidizing agent with various industrial applications. Gastrointestinal exposure, whether by ingestion or rectal administration, can lead to severe local toxicity, gastrointestinal ischemia, and even gas embolism. Despite these risks, H_2_O_2_ is sometimes used in naturopathic and alternative medicine for self-treating constipation.^1^

## CASE REPORT

A 56-year-old man with no significant medical history presented with complaints of generalized abdominal pain and rectal bleeding that began shortly after self-administering a hydrogen peroxide enema for constipation relief. The patient used a 1:10 dilution of hydrogen peroxide, prepared by mixing 6 oz of hydrogen peroxide with 60 oz of water. Within minutes of administering the enema, he developed severe abdominal cramping and rectal pain, followed by multiple bowel movements mixed with small amounts of fresh, bright red blood. There was no associated fever, chills, nausea, or vomiting.

**Figure 1.**
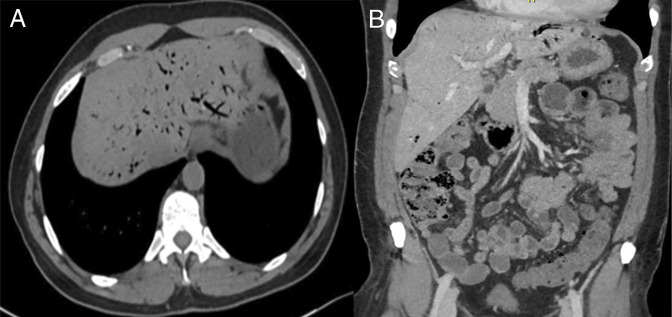
Axial computed tomography image of the abdomen demonstrating extensive portal venous gas in both liver lobes (A). Coronal view showing multiple tubular gas locules in the sigmoid mesocolon, consistent with mesenteric venous gas (B).

On presentation, his vital signs were stable. Physical examination revealed generalized abdominal tenderness without guarding or rigidity. Initial laboratory studies were notable for an elevated white blood cell count (14.22 × 10^3^/μL) and normal anion gap metabolic acidosis (Table 1). An abdominal computed tomography scan (Figure 1) revealed extensive portal venous gas in both liver lobes, tubular gas locules in the sigmoid mesocolon, and diffuse colonic wall thickening from the hepatic flexure to the rectum. Arterial vasculature was patent.

**Table 1.** Initial laboratory results

	Value	Reference range
White blood cells (×10^3^/μL)	14.22	4.4–10.5
Hemoglobin (g/dL)	14.2	12.6–16.7
Platelets (×10^3^/μL)	239	139–361
Sodium (mmol/L)	134	135–145
Potassium (mmol/L)	4.7	3.5–5.0
Chloride (mmol/L)	102	98–110
Bicarbonate (mmol/L)	20.0	22.0–26.0
Creatinine (mg/dL)	0.82	0.6–1.2
BUN (mg/dL)	22.0	5.0–25.0
ALT (U/L)	15	4–51
AST (U/L)	30	5–46
Total bilirubin (mg/dL)	0.30	0.10–1.50
ALP (U/L)	63	40–129
Lipase (U/L)	22	10–60
Lactic acid (mmol/L)	0.9	0.5–1.90
CRP (mg/L)	12	<5

ALP, alkaline phosphatase; ALT, alanine aminotransferase; AST, aspartate aminotransferase; BUN, blood urea nitrogen; CRP, C-reactive protein.

Based on these findings, the patient was diagnosed with portal and mesenteric venous air embolism, along with chemical colitis secondary to a H_2_O_2_ enema. He was placed in the Trendelenburg position and received a single session of hyperbaric oxygen (HBO) therapy at 2.8 atm absolute for 2.5 hours.

**Figure 2.**
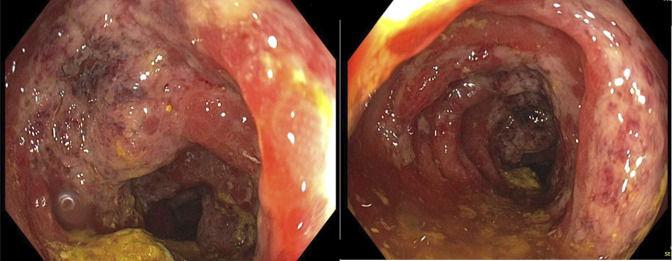
Colonoscopy showing diffuse mucosal congestion, friability, erythema, and ulceration consistent with severe acute colitis.

Colonoscopy was performed to further characterize and assess the extent of colitis, using the water immersion technique to minimize colonic distension and reduce perforation risk. Colonoscopy revealed diffuse areas of severely altered vasculature with congested, erythematous, friable, and ulcerated mucosa throughout the entire colon (Figure 2). Colonic biopsies confirmed acute colitis. The ascending colon showed mild acute inflammation, while the transverse colon demonstrated gland dropout, ulceration, and lamina propria hyalinization. The descending colon revealed mucosal injury with ulceration, crypt dilation and atrophy, and superficial mucus, fibrin, and inflammatory cells. No dysplasia or malignancy was identified.

**Figure 3.**
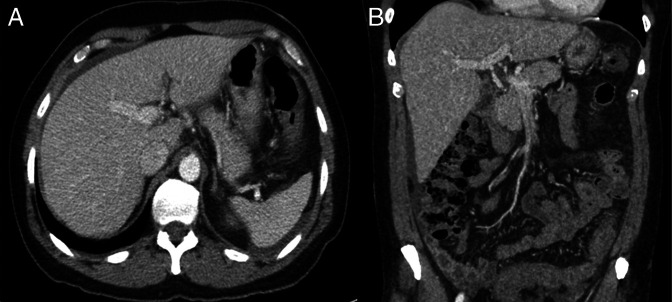
Axial computed tomography image of the abdomen demonstrating resolution of portal venous gas (A) and coronal view showing resolution of mesenteric venous gas (B) following hyperbaric oxygen therapy.

The patient was managed conservatively with intravenous fluids, analgesia, and empiric piperacillin-tazobactam. Follow-up abdominal computed tomography performed 48 hours postcolonoscopy showed complete resolution of portal venous gas and air along the sigmoid mesocolon but persistent colitis extending from the distal transverse colon to the rectum (Figure 3). Symptoms fully resolved shortly thereafter, and the patient was discharged with a 5-day course of amoxicillin-clavulanate and outpatient gastroenterology follow-up.

## DISCUSSION

This case highlights some of the potential complications of hydrogen peroxide enema, including venous air embolism and chemical colitis. Hydrogen peroxide causes tissue damage by 3 mechanisms: direct caustic injury leading to mucosal erosion, lipid peroxidation of cell membranes, and formation of oxygen gas.^2^ This latter effect occurs through an exothermic reaction (2H_2_O_2_ → 2H_2_O + O_2_ + heat) catalyzed by endogenous catalase in mucous membranes, releasing large volumes of oxygen. As little as 30 mL of 35% hydrogen peroxide can liberate up to 3.5 L of oxygen.^3^ If gas volume exceeds blood solubility, it may lead to vascular gas emboli, mechanical distension, and perforation.^4,5^

H_2_O_2_ is often erroneously promoted in alternative medicine as a treatment of constipation, cancer, and bacterial overgrowth.^1^ Several case reports describe portal venous gas from ingestion and chemical colitis from enemas.^1,6,7^ However, our case is unique in that portal venous gas embolism resulted from an H_2_O_2_ enema. Oxygen bubbles likely entered submucosal venules through damaged mucosal capillaries, reaching the mesenteric venous system and portal vein. To our knowledge, only 3 such cases are reported. Two cases reported sudden symptom onset with resolution within 24–48 hours, while the third case recovered fully after HBO therapy.^8–10^

The clinical presentation of hydrogen peroxide toxicity varies by route, concentration, and volume of exposure. Ingestion typically causes acute gastrointestinal symptoms such as nausea, vomiting, abdominal pain, and hematemesis due to caustic injury and gas expansion. Rectal administration may lead to bloody diarrhea, tenesmus, and perianal pain from chemical colitis or proctitis. Symptoms usually appear within minutes to hours. Complications such as pneumatosis intestinalis, ischemic colitis, or perforation may occur, requiring clinical vigilance.^2^ Stroke, myocardial infarction, and obstructive shock have also been reported, with systemic arterial embolism carrying high morbidity and mortality.^11,12^ A 10-year review of 294 ingestion cases found embolic events in 13.9% and death or disability in 6.8%.^13^ A systematic review of 126 cases showed embolic events typically occur within 10 hours.^14^

Current evidence for HBO therapy in air embolism is largely based on observational data and mechanistic rationale. HBO reduces bubble size through Boyle law and increases plasma oxygen solubility per Henry law, aiding gas resorption and elimination. The European Consensus Conference on Hyperbaric Medicine gives a level C recommendation for HBO in gas embolism, reflecting limited evidence.^15^ In arterial gas embolism, early HBO has been linked to improved neurological outcomes and lower mortality.^6,14^ Its role in isolated venous gas embolism is less defined. Case series suggest lower rates of subsequent embolic events in patients with portal venous gas treated with HBO vs conservative management, though successful conservative outcomes have also been reported.^14,16^

Although portal venous gas emboli may resolve spontaneously, the timeline and associated patient risks during this period remains uncertain. Given the high morbidity and mortality of secondary arterial embolism and HBO's low-risk profile, HBO may be a reasonable treatment for venous air embolism, especially in cases with large gas burden, suspected paradoxical embolism, or uncertain arterial involvement. A single session may resolve portal venous gas, potentially avoiding intensive care unit admission or repeat imaging.

In conclusion, this case adds to the growing literature on a rare but serious condition, highlighting the need for public education on the risks of unapproved hydrogen peroxide use and the importance of individualized management, including HBO therapy for embolic or systemic complications.

## DISCLOSURES

Author contributions: P. Mann: Initial idea, Designed the case report, performed literature review, drafted the initial manuscript and revised the final version. T. Alsaleh: Helped with literature review, review and editing of the manuscript. N. Shafique: Helped with literature review, review and editing of the manuscript. M.K. Almujarkesh: Assisted in endoscopic evaluation and image acquisition, provided insight into the case discussion and revised the manuscript. B.A. Bani Fawwaz: Assisted in endoscopic evaluation and image acquisition, provided insight into the case discussion and revised the manuscript. I. Inayat: Provided senior supervision, contributed to critical revisions and approved the final manuscript for submission and is the article guarantor.

Financial disclosure: None to report.

Informed consent was obtained for this case report.

## ABBREVIATIONS:

ALP: alkaline phosphatase
ALT: alanine aminotransferase
AST: aspartate aminotransferase
BUN: blood urea nitrogen
CRP: C-reactive protein
CT: computed tomography
H_2_O_2_: hydrogen peroxide
HBO: hyperbaric oxygen
ICU: Intensive Care Unit
O_2_: oxygen
U/L: units per liter
WBC: white blood cell
